# Senescent Cell Derived Artificial Vesicle‐Based Senolytic Sonovaccine Platform with Augmented Lymph Node Delivery and Antigen Cross‐Presentation Efficacy

**DOI:** 10.1002/advs.75770

**Published:** 2026-05-20

**Authors:** Liang Zhang, Yubo Lai, Jia Wang, Lingling Liu, Jieyuan An, Mi Qu, Yuan Liang, Bijun Tan, Guodong Yang, Xuekang Yang, Lijun Yuan

**Affiliations:** ^1^ Department of Ultrasound Medicine Tangdu Hospital The Fourth Military Medical University Xi'an Shaanxi China; ^2^ Department of Burns and Cutaneous Surgery Xijing Hospital The Fourth Military Medical University Xi'an Shaanxi China; ^3^ State Key Laboratory of Holistic Integrative Management of Gastrointestinal Cancers Department of Biochemistry and Molecular Biology The Fourth Military Medical University Xi'an Shaanxi China

**Keywords:** atherosclerosis, cross‐presentation, lymph node targeting, senescent cell derived artificial vesicle, senolytic vaccine

## Abstract

The efficacy of senolytic vaccines aiming to clear senescent cells is limited by narrow antigen coverage and inefficient CD8^+^ T cell priming due to poor lymph node (LN) accumulation and inefficient antigen cross‐presentation. Here, we report SenoVac, a modular dual‐component nanovaccine platform explicitly consisting of two sequentially administered key components: an “albumin hitchhiking” DSPE‐PEG‐DBCO conjugate, and azide‐functionalized senescent cell‐derived artificial vesicles (SCAVs) that co‐encapsulate the sonosensitizer hematoporphyrin monomethyl ether (HMME) and the TLR7/8 agonist adjuvant resiquimod (R848). The first administered DSPE‐PEG‐DBCO conjugate leverages endogenous albumin transport to traffic efficiently to LNs, where it installs bioorthogonal DBCO docking sites; the subsequent administration of SenoVac's azide‐functionalized SCAVs facilitates specific and sustained accumulation in LNs via click chemistry. In the LNs, local therapeutic ultrasound irradiation activates HMME to generate reactive oxygen species (ROS), which disrupt dendritic cell endosomal membranes, promoting senescence‐associated antigens (SAAs) escape into the cytoplasm and robust cross‐presentation to CD8^+^ T cells. In an ApoE^−/−^ mouse model of atherosclerosis, this integrated platform effectively cleared plaque senescent cells, attenuated disease progression, and showed a favorable safety profile. Our work establishes a translatable strategy that combines active LN targeting with stimulus‐responsive cross‐presentation enhancement for senolytic immunotherapy.

## Graphical abstract

A senolytic sonovaccine platform (SenoVac) is developed, in which senescent cell‐derived vesicles serve as broad senescent cell antigen reservior. The “2‐step” click chemistry strategy for effective lymph node delivery, and ultrasound‐triggered endosomal escape to boost cross‐presentation, ensures efficient senescent cell clearance and disease attenuation in vivo.

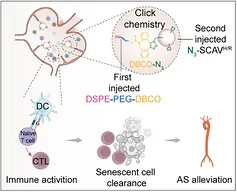

## 1 Introduction

Vaccinology has evolved beyond infectious disease prevention, emerging as a transformative therapeutic strategy for conditions like cancer and, more recently, cellular senescence [1, 2, 3]. Senolytic vaccines constitute a novel class of immunotherapies designed to direct the adaptive immune system to identify and clear senescent cells, offering a promising approach to treat age‐associated diseases such as atherosclerosis (AS) [4, 5, 6].

Several senescence‐associated antigens (SAAs) have been identified as potential targets, including CD153 [7], glycoprotein non‐metastatic melanoma protein B (GPNMB) [8], urokinase‐type plasminogen activator (uPAR) [9, 10], dipeptidyl‐peptidase 4 (DPP4) [11], beta‐2 microglobulin (B‐2 M) [12], and ligands for natural killer group 2 member D (NKG2D) [13], among others. Proof‐of‐concept vaccines targeting CD153 and GPNMB have demonstrated efficacy in clearing senescent cells and eliciting associated therapeutic benefits [7, 8]. However, owing to the pronounced heterogeneity of senescent cells, vaccines directed against a single antigen are unlikely to cover the majority of the senescent population [14]. In vitro‐induced senescent cells express a broad spectrum of senescence‐associated proteins, providing a diverse antigenic source for vaccine design [15]. Senescent cell derived artificial vesicles (SCAVs) preserve a parental‐cell‐like proteome, encapsulating both cytosolic and membrane‐bound antigens, which makes them an ideal carrier platform for whole‐cell‐based senolytic vaccines with broad antigen coverage [16, 17, 18].

Effective clearance of senescent cells depends on robust CD8^+^ cytotoxic T lymphocyte (CTL) activation [19]. This process is orchestrated primarily in lymph node (LN), where dendritic cells (DCs) and other antigen‐presenting cells (APCs) present antigen to naïve T cells [20]. Thus, the efficacy of a vaccine is fundamentally contingent upon the targeted delivery of its antigens to LNs [21, 22]. Passive lymphatic drainage serves as an efficient route for targeted delivery to LNs, with its effectiveness being strongly size‐dependent [23, 24, 25]. Unlike smaller (<10 nm) particles that enter the bloodstream or larger (>100 nm) ones that undergo slower APC‐dependent transport, particles ranging from 10 to 100 nm diffuse most efficiently through inter‐endothelial gaps into initial lymphatic capillaries, establishing this as the optimal size range for passive lymphatic targeting [25]. However, passive drainage alone often leads to inadequate retention in LNs, shortening the window for antigen capture and processing by resident APCs [26]. A further challenge lies in the antigen‐presentation pathway: exogenous protein antigens are typically channeled into the major histocompatibility complex class II (MHC‐II) pathway [27]. To prime CD8^+^ CTLs, antigens must instead undergo cross‐presentation, a process limited by inefficient escape of internalized antigen from endo‐lysosomal compartments into the cytosol [28, 29]. Thus, developing a nanovaccine system that combines targeted LN accumulation with enhanced endo‐lysosomal escape remains a critical unmet need.

**SCHEME 1:**
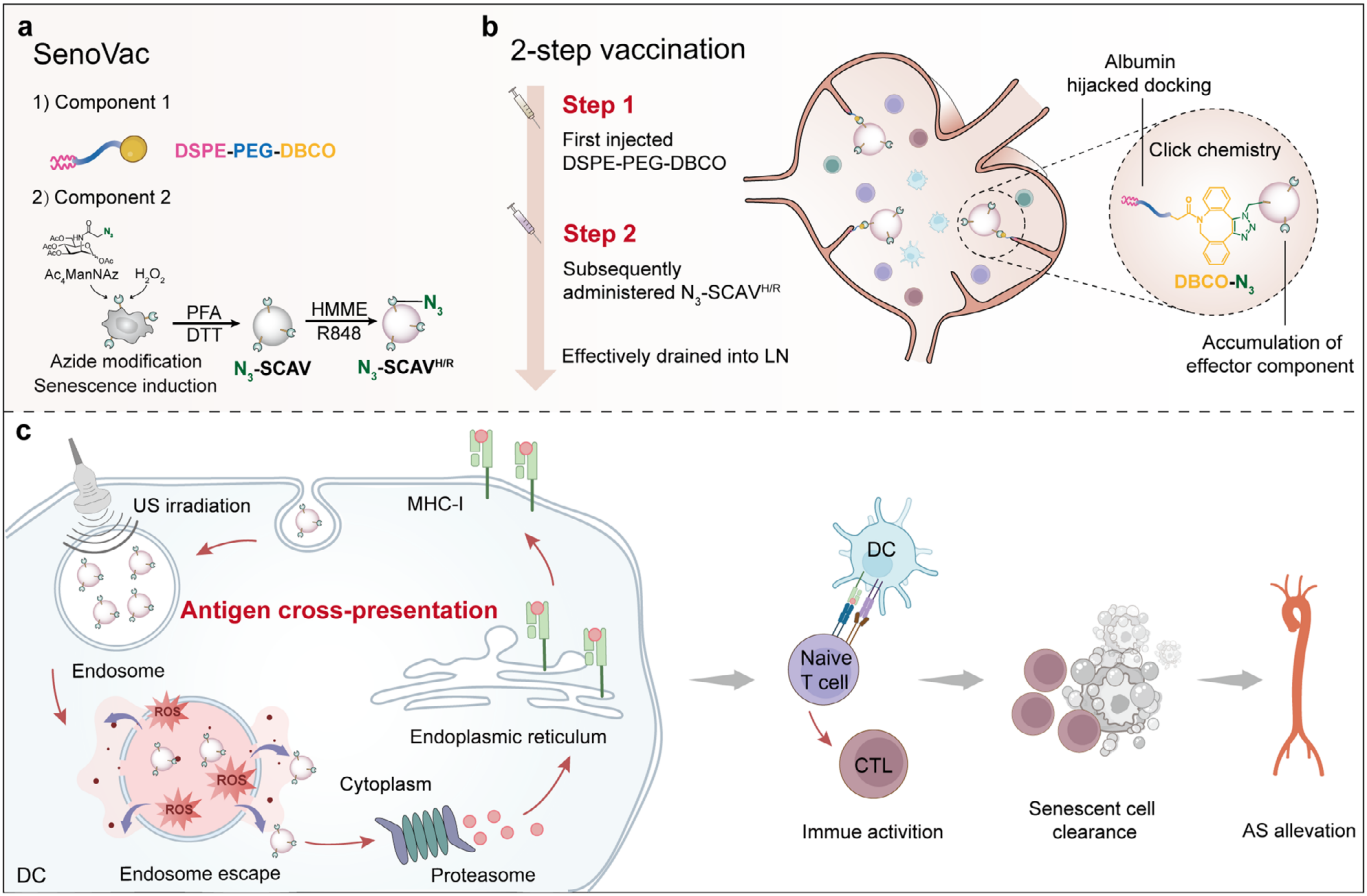
Construction and function of SCAVs‐based senolytic sonovaccine. (a) The dual‐component system comprises a pre‐targeting DSPE‐PEG‐DBCO conjugate and antigen‐presenting SCAVs loaded with HMME and R848 (N_3_‐SCAV^H/R^). (b) Sequential administration strategy exploiting in vivo click chemistry for specific LN retention of N_3_‐SCAV^H/R^. (c) US‐triggered sonodynamic action disrupts DC endosomes, potentiating cross‐presentation to CD8^+^ T cells for efficient senescent cell clearance and attenuation of AS.

Here, we report SenoVac, a modular dual‐component sonovaccine platform explicitly consisting of two sequentially administered key components: an “albumin hitchhiking” DSPE‐PEG‐DBCO conjugate, and azide‐functionalized SCAVs which co‐encapsulate the sonosensitizer hematoporphyrin monomethyl ether (HMME) and the TLR7/8 agonist adjuvant resiquimod (R848). Briefly, SCAVs component inherently carries a broad repertoire of native SAAs, a critical advantage over single‐antigen vaccines. Moreover, SenoVac's dual‐component design enables efficient LN delivery and retention. The first administered DSPE‐PEG‐DBCO conjugate leverages endogenous albumin transport to traffic efficiently to LNs, where it installs bioorthogonal DBCO docking sites; the subsequent administration of SenoVac's azide‐functionalized SCAVs facilitates specific and sustained accumulation in LNs via click chemistry‐mediated anchoring to these pre‐installed sites. In the LNs, local therapeutic ultrasound (US) irradiation then activates HMME within the SCAVs to generate reactive oxygen species (ROS), which disrupt DCs’ endosomal membranes, promoting antigen escape into the cytoplasm and robust cross‐presentation to CD8^+^ T cells (Scheme 1). In an ApoE^−/−^ mouse model of AS, this integrated platform effectively cleared plaque senescent cells, attenuated disease progression, and showed a favorable safety profile. Our work establishes a translatable strategy that combines active LN targeting and stimulus‐responsive cross‐presentation enhancement for senolytic immunotherapy.

## 2 Results

### 2.1 Preparation of Cell‐Derived Artificial Vesicles with Effective LN Targeting

Cell‐derived artificial vesicles (CAVs) were generated via a sulfhydryl‐blocking chemical induction method, which triggers controlled cell blebbing to produce nanosized vesicles at high yield [30]. To obtain CAVs suitable for lymphatic drainage, we first optimized the induction conditions. HEK293T cells were treated with sulfhydryl‐blocking reagents containing varying concentrations of paraformaldehyde (PFA). The results indicated that vesicle size increased with PFA concentration. A formulation containing 25 mm PFA yielded CAVs with a uniform hydrodynamic diameter below 100 nm, ideal for passive lymphatic transport, and was therefore selected for all subsequent preparations (Figure S1).

**FIGURE 1:**
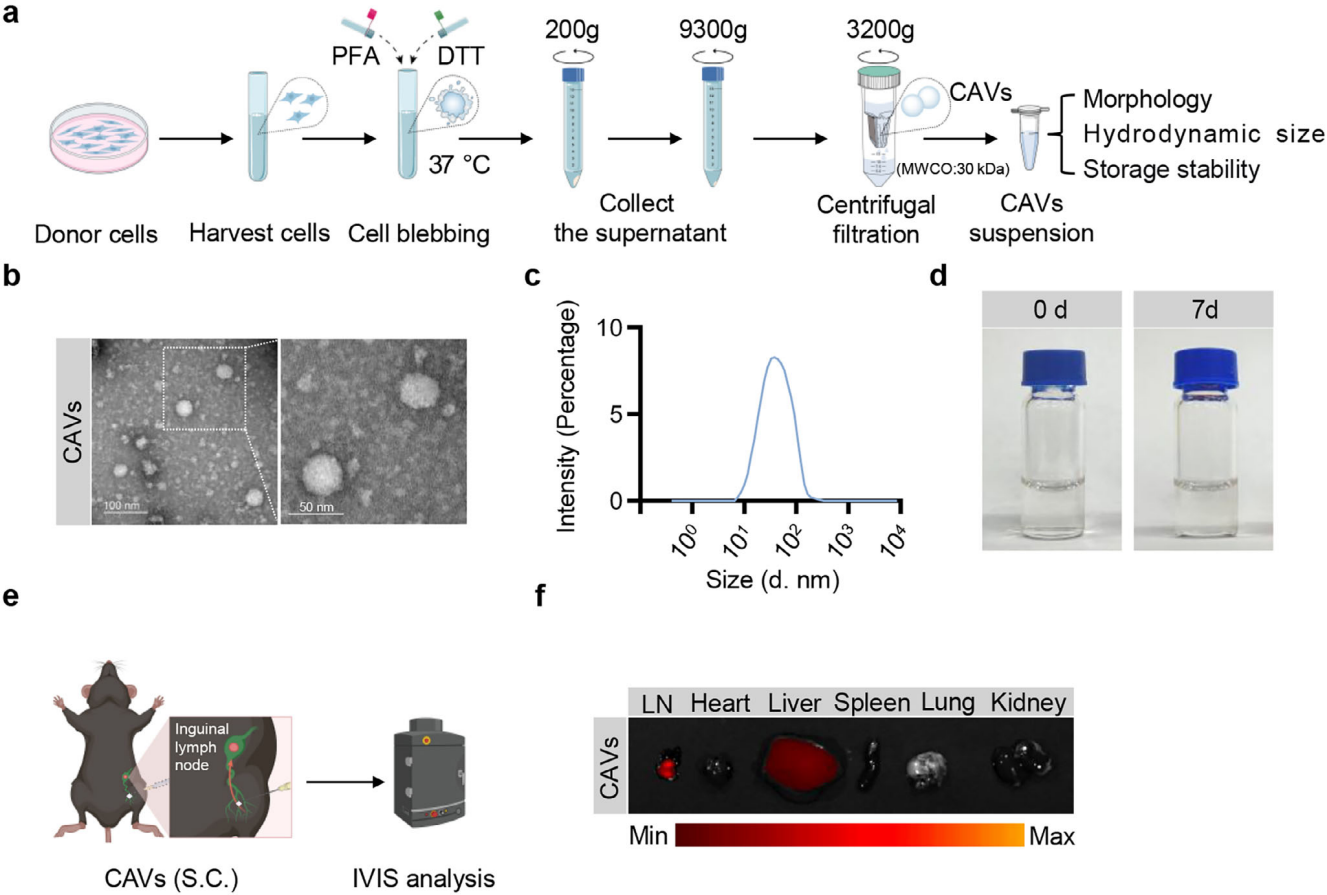
Preparation and characterization of CAVs for lymphatic drainage. (a) Schematic illustrating the preparation steps of CAVs. Molecular weight cut‐off (MWCO) = 30 kDa. (b) Representative TEM image of CAVs. (c) Representative hydrodynamic size distribution of CAVs. (d) Representative digital images of CAVs before and after 7 d of storage at 4°C. (e) Schematic of the in vivo lymphatic drainage assay monitored by in vivo imaging system (IVIS). (f) Representative IVIS image of DiR‐labeled CAVs distribution in dLNs and other major organs at 24 h post‐subcutaneous injection.

CAVs were then produced under the optimized condition (25 mm PFA and 2 mm dithiothreitol (DTT) for 2 h) and systematically characterized (Figure 1a). Transmission electron microscopy (TEM) revealed that CAVs exhibited a uniform spherical morphology (Figure 1b). Dynamic light scattering (DLS) analysis confirmed a narrow hydrodynamic size distribution below 100 nm (Figure 1c). Furthermore, the CAVs suspension remained clear and stable without visible aggregation or precipitation after 7 days of storage at 4°C. DLS analysis further revealed no significant change in hydrodynamic size over the same period, demonstrating excellent colloidal stability (Figure 1d; Figure S2). However, following subcutaneous administration, in vivo imaging revealed that while DiR‐labeled CAVs did undergo lymphatic drainage to LNs, the fluorescence signal in the LNs was only marginally higher than that in the liver, indicating suboptimal LN accumulation efficiency (Figure 1e,f).

**FIGURE 2:**
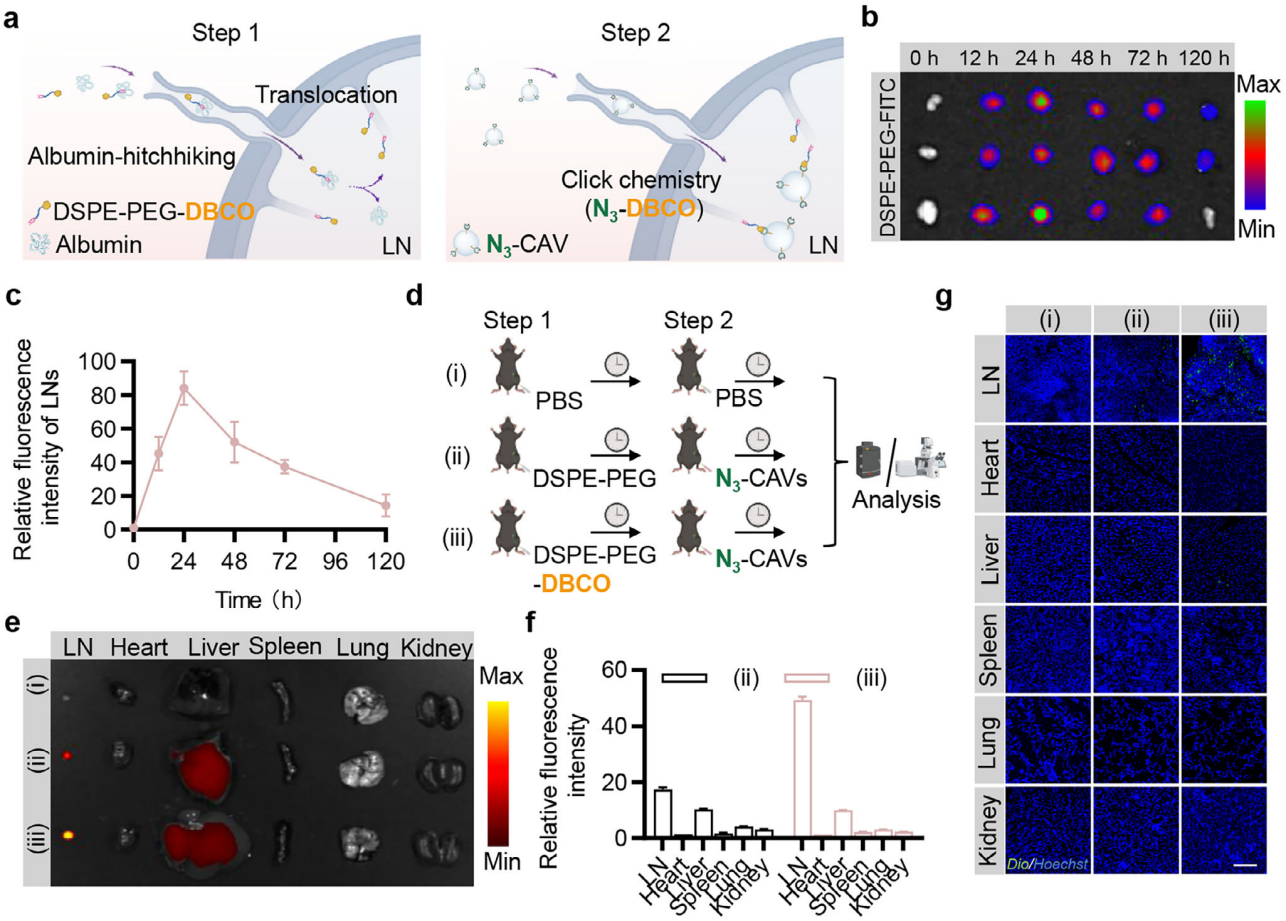
Design and characterization of the modular dual‐component LN‐targeted aggregation system based on click chemistry. (a) Scheme illustrating the working mechanism of the modular dual‐component LN‐targeted aggregation platform: following subcutaneous injection, DSPE‐PEG‐DBCO conjugate leverages endogenous albumin transport to traffic efficiently to LNs, where it installs bioorthogonal DBCO docking sites; the subsequent administration of N_3_‐CAVs facilitates specific and sustained accumulation in LNs via click chemistry‐mediated anchoring to these pre‐installed sites. (b) IVIS imaging of isolated inguinal LNs at various time points post DSPE‐PEG‐FITC injection. (c) Quantification of accumulated fluorescence in LNs over time, based on data from (b). (d) Schematic of the in vivo procedure for the modular LN‐targeted aggregation system based on click chemistry. (e) Representative IVIS analysis of DiR‐labeled N_3_‐CAVs distribution in LNs and major organs under the indicated pre‐treatments. (f) Relative fluorescence density in LNs and major organs under different treatments, quantified from (e). (g) Representative confocal laser scanning microscopy (CLSM) image of DiO‐labeled N_3_‐CAVs distribution in LNs and major organs under the indicated pre‐treatments. Scale bar = 200 µm.

To achieve active and efficient LN targeting, we designed a modular two‐component system based on click chemistry. Capitalizing on albumin's inherent LN‐homing ability, we synthesized DSPE‐PEG‐DBCO to serve as an LN‐pre‐targeting anchor via “albumin hitchhiking”, with its terminal DBCO group providing a bioorthogonal docking sites. The complementary azide group was introduced onto CAVs through metabolic glycan engineering, yielding N_3_‐CAVs (Figures S3 and S4). This design enables N_3_‐CAVs to accumulate in LNs via click chemistry anchoring to pre‐installed DBCO sites (Figure 2a).

The LN homing of the pre‐targeting component was first validated. FITC‐labeled DSPE‐PEG was subcutaneously injected into C57BL/6 mice, and its dLNs distribution was monitored via IVIS. Quantitative analysis of LNs fluorescence intensity revealed a peak at 24 h post‐injection, followed by a gradual decline. Significant fluorescence was retained at 72 h and remained detectable at 120 h (Figure 2b,c). These results demonstrate effective and sustained LN labeling by DSPE‐PEG‐DBCO, supporting its potential as a stable docking site for subsequent anchoring of N_3_‐CAVs.

The bioorthogonal reactivity between DBCO‐anchored sites and N_3_‐CAVs was examined in vitro. SVEC4‐10 cells pre‐incubated with an albumin‐DSPE‐PEG‐DBCO complex showed significantly higher retention of DiI‐labeled N_3_‐CAVs than controls, demonstrating specific click‐mediated conjugation (Figure S5). We next assessed the delivery efficiency of the modular system in vivo. Mice pretreated with DSPE‐PEG‐DBCO and subsequently injected in the footpad with DiR‐labeled N_3_‐CAVs exhibited approximately threefold higher LN fluorescence than the DSPE‐PEG‐pretreated control group, demonstrating enhanced LN delivery and retention via pre‐installed DBCO docking sites (Figure S6). Biodistribution analysis further demonstrated targeting specificity. After subcutaneous pretreatment with DSPE‐PEG‐DBCO and subsequent N_3_‐CAV administration, strong fluorescence localized predominantly in dLNs, with moderate liver uptake and minimal signal in other major organs (heart, spleen, lung, kidney) (Figure 2d–f). Confocal imaging corroborated this LN‐specific distribution (Figure 2g). Collectively, these results validate that this modular dual‐component design enables efficient LN delivery and retention.

### 2.2 Preparation of US‐Responsive CAVs to Enhance Cross‐Presentation

**FIGURE 3:**
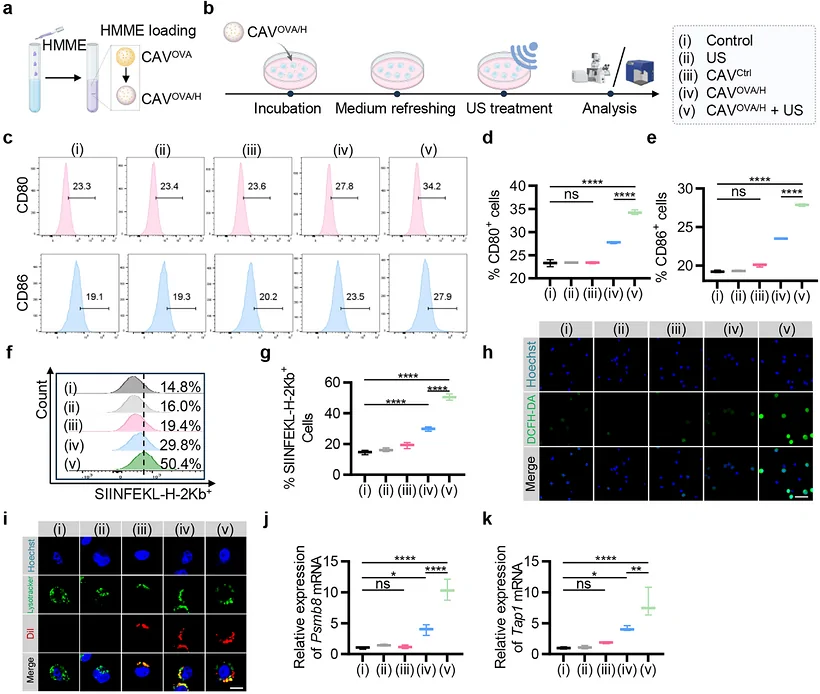
Preparation of US‐responsive CAVs to enhance cross‐presentation. (a) Schematic of the CAV^OVA/H^ preparation process. (b) Schematic diagram showing the experimental procedure of DC maturation analysis. (c) Representative flow cytometry data of DC maturation markers. (d) The percentage of CD80^+^ DCs as quantified from the data in (c). (e) The percentage of CD86^+^ DCs as quantified from the data in (c). (f) Representative flow cytometry data of SIINFEKL peptide presented by DCs. (g) The percentage of SIINFEKL‐H‐2Kb^+^ DCs as quantified from the data in (f). (h) CLSM images of ROS generated in DCs under the indicated treatments. Nuclei are counterstained with Hoechst (blue). Scale bar = 50 µm. (i) CLSM images showing the colocalization of DiI‐labeled CAVs (red) with lysosomes (green) in DCs. Nuclei are counterstained with Hoechst (blue). Scale bar = 10 µm. (j) qPCR analysis of *Psmb8* mRNA expression in DCs under the indicated treatments. (k) qPCR analysis of *Tap1* mRNA expression in DCs under the indicated treatments. Data are expressed as mean ± SEM. Statistical significance was determined by one‐way ANOVA with Tukey's post hoc test. ^*^ *p <* 0.05, ^**^ *p <* 0.01, ^****^ *p* < 0.0001, ns, not significant.

To evaluate the immunostimulatory potential of CAVs as a vaccine carrier, ovalbumin (OVA) was selected as a model antigen. OVA antigen‐loaded CAVs, generated from OVA‐transfected HEK293T cells, were termed CAV^OVA^ (Figure S7a,b). qPCR analysis confirmed successful OVA loading, as evidenced by a significant upregulation of *Ova* mRNA in both the transfected donor cells and the isolated CAV^OVA^ (Figure S7c,d). Furthermore, the sonosensitizer HMME was incorporated into the CAV^OVA^ membrane via co‐incubation, resulting in the construction of US‐responsive CAV^OVA/H^ (Figure 3a).

The uptake of antigens by APCs is a critical determinant of the ensuing immune response. Therefore, the uptake of CAVs by DCs was evaluated first. CLSM analysis revealed that DiI‐labeled CAV, CAV^OVA^, and CAV^OVA/H^ were all efficiently internalized by DCs after co‐incubation, indicating that OVA expression and HMME loading did not alter their cellular internalization behavior (Figure S8). Subsequently, the effect of CAV^OVA/H^ in combination with US irradiation on DCs maturation and antigen presentation was assessed (Figure 3b). Flow cytometry analysis demonstrated that CAV^OVA/H^ together with US treatment significantly upregulated the expression of the co‐stimulatory molecules CD80 and CD86 on DCs, suggesting effective promotion of DCs maturation (Figure 3c–e). Moreover, the proportion of DCs presenting the SIINFEKL‐H‐2Kb complex was markedly higher in the CAV^OVA/H^ ‐treated group compared to controls, and increased further upon US irradiation, indicating that sonodynamic treatment effectively enhanced cross‐presentation efficiency (Figure 3f,g).

Given that ROS are critical signaling molecules that regulate cellular processes and that moderate oxidative stress can potentiate antigen processing and presentation in DCs, intracellular ROS levels were examined to elucidate the mechanism of enhanced cross‐presentation by CAV^OVA/H^ under US stimulation. CLSM analysis revealed that while neither CAV^Ctrl^ nor non‐sonicated CAV^OVA/H^ altered intracellular ROS levels, US irradiation of CAV^OVA/H^ significantly augmented ROS production (Figure 3h). Concurrently, we observed that its subcellular localization was notably altered: similar to CAV^Ctrl^, non‐sonicated CAV^OVA/H^ co‐localized with lysosomes, but upon US irradiation, it dissociated from them, suggesting triggered endo/lysosomal escape likely attributable to ROS‐induced lysosomal membrane destabilization (Figure 3i). Further qPCR analysis revealed that CAV^OVA/H^ upon US irradiation upregulated the expression of proteasome subunit gene *Psmb8* and endoplasmic reticulum antigen transporter *Tap1*, suggesting that, in addition to facilitating endosomal escape, ROS may also promote antigen processing by activating the MHC‐I antigen presentation pathway (Figure 3j,k). These findings demonstrate that, upon DC internalization, CAV^OVA/H^ enters the endo/lysosomal system; subsequent sonodynamic stimulation triggers ROS production, which not only disrupts endosomal membranes to enable antigen escape into the cytosol but also may be involved in the regulation of antigen processing‐related gene expression, thereby enhancing cross‐presentation and potentiating a more effective cytotoxic T‐cell response.

### 2.3 LN‐Targeted Aggregation System Combined with US Irradiation Augments Immune Activation In Vivo

**FIGURE 4:**
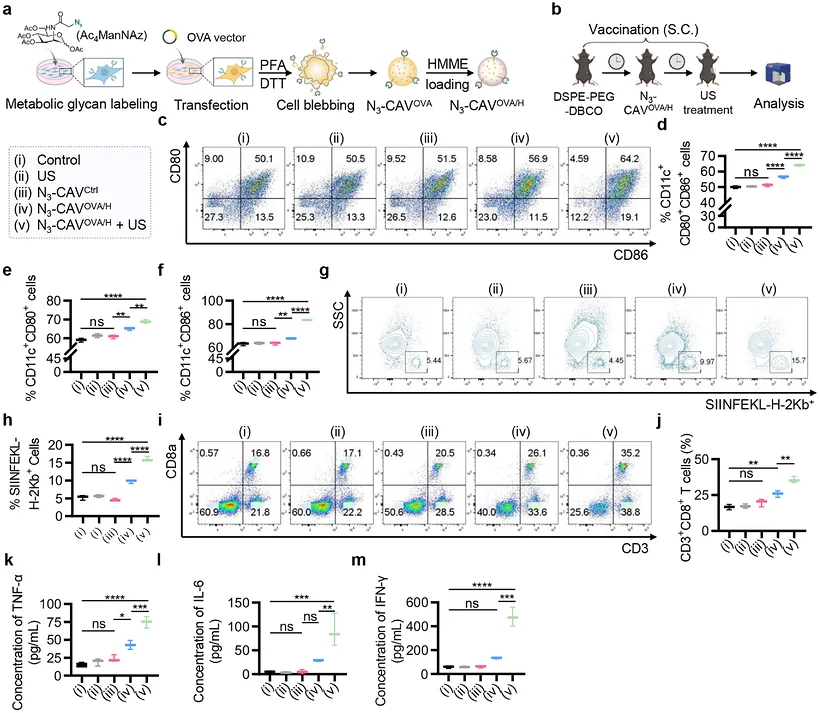
LN‐targeted aggregation system combined with US irradiation augments immune activation in vivo. (a) Schematic of the preparation procedure for N_3_‐CAV^OVA/H^. (b) Schematic of the in vivo vaccination procedure using N_3_‐CAV^OVA/H^. (c) Representative flow cytometric analysis of DC maturation markers in dLNs of C57BL/6 mice at 3 d post indicated treatments. (d‐f) Quantification of the percentages of CD11c^+^CD80^+^CD86^+^ (d), CD11c^+^CD80^+^ (e), and CD11c^+^CD86^+^ (f) DC populations from (c). (g) Representative flow cytometric analysis of SIINFEKL‐H‐2Kb presentation by DCs in vaccine dLNs of C57BL/6 mice at 3 d post the indicated treatments. (h) Quantification of the percentage of SIINFEKL‐H‐2Kb^+^ DCs from (g). (i) Flow cytometry analysis of CTLs in vaccine dLNs across treatment groups on day 10. (j) Quantification of the percentage of CTLs from (i). (k–m) Serum levels of TNF‐α (k) and IL‐6 (l) at 3 d, and IFN‐γ (m) at 10 d post‐treatment. Data are expressed as mean ± SEM. Statistical significance was determined by one‐way ANOVA with Tukey's post hoc test. ^**^ *p* < 0.01, ^***^ *p* < 0.001, ^****^ *p* < 0.0001, ns, not significant.

Building on in vitro evidence that CAV^OVA/H^ promotes DCs cross‐presentation under US stimulation, we employed the modular LN‐targeted aggregation system to enhance its targeted delivery to LNs in vivo and evaluate its efficiency in activating cellular immunity. To construct the vaccine, donor cells were engineered through metabolic glycan labeling and OVA vector transfection, followed by sulfhydryl‐blocking chemical induction to generate N_3_‐CAV^OVA^. The sonosensitizer HMME was subsequently loaded into the membrane, yielding the final vaccine construct, N_3_‐CAV^OVA/H^ (Figure 4a). C57BL/6 mice were subcutaneously pretreated with DSPE‐PEG‐DBCO and, 24 h later, vaccinated with N_3_‐CAV^OVA/H^ followed by US irradiation. Immune activation was assessed at multiple time points post‐vaccination (Figure 4b).

Given previous reports linking LN expansion to immune activation, dLNs were collected and their size measured at various time points [31]. At day 3, dLNs from N_3_‐CAV^OVA^‐treated group exhibited increases in size and weight compared with the control, with the most pronounced effect observed when combined with US. In contrast, US‐alone did not induce such changes (Figure S9). Super‐resolution US imaging performed before and 3 days after vaccination revealed that US‐augmented vaccination significantly enhanced several key hemodynamic parameters within the dLNs, including mean velocity, perfusion index, vascular ratio, and complexity level. In contrast, parameters such as mean density and curvature index remained unchanged. These data indicate a robust immune response to the vaccination (Figure S10). The temporal dynamics of dLNs expansion were further monitored via serial B‐mode US imaging. The N_3_‐CAV^OVA^ + US group displayed maximal enlargement on day 3, followed by a gradual regression (Figure S11). Consistently, by day 10, differences in dLN size and weight were no longer statistically significant (Figure S12). These findings demonstrate that N_3_‐CAV^OVA/H^ combined with US stimulation, effectively induces structural and hemodynamic responses in dLNs.

Flow cytometric analysis of dLNs revealed that on day 3 post‐vaccination, DCs from N_3_‐CAV^OVA/H^‐immunized mice exhibited upregulated expression of the costimulatory molecules CD80 and CD86. This effect was further enhanced by US stimulation, suggesting sonodynamic potentiation of DC maturation (Figure 4c–f). This effect subsided by day 10 (Figure S13). Next, we also evaluated MHC‐I‐restricted antigen presentation on day 3. The proportion of SIINFEKL‐H‐2Kb complex‐positive DCs was modestly elevated in the N_3_‐CAV^OVA/H^ group and significantly higher in the US‐augmented group, indicating enhanced cross‐presentation efficiency (Figure 4g,h).

Analysis of CD8^+^ T cell activation in dLNs revealed minimal activation on day 3 (Figure S14). However, by day 10, the proportion of activated CD8^+^ T cells was markedly increased in the N_3_‐CAV^OVA/H^ group and further elevated in the group that also received US treatment (Figure 4i,j). Serum cytokine analysis revealed that US‐augmented vaccination induced transient elevations in TNF‐α and IL‐6 levels at day 3, which returned to baseline by day 10 (Figure 4k,l; Figure S15a,b). In contrast, IFN‐γ levels in the N_3_‐CAV^OVA/H^ + US group showed a modest increase on day 3 but a marked increase on day 10, indicating progressive activation of adaptive immunity (Figure S15c; Figure 4m). These data demonstrate that the modular LN‐targeted delivery system, when combined with US stimulation, robustly enhances DC maturation, antigen cross‐presentation, CD8^+^ T cell activation, and cytokine responses, leading to potent antigen‐specific cellular immunity.

### 2.4 Preparation and Characterization of SCAV‐Based Senolytic Sonovaccine

**FIGURE 5:**
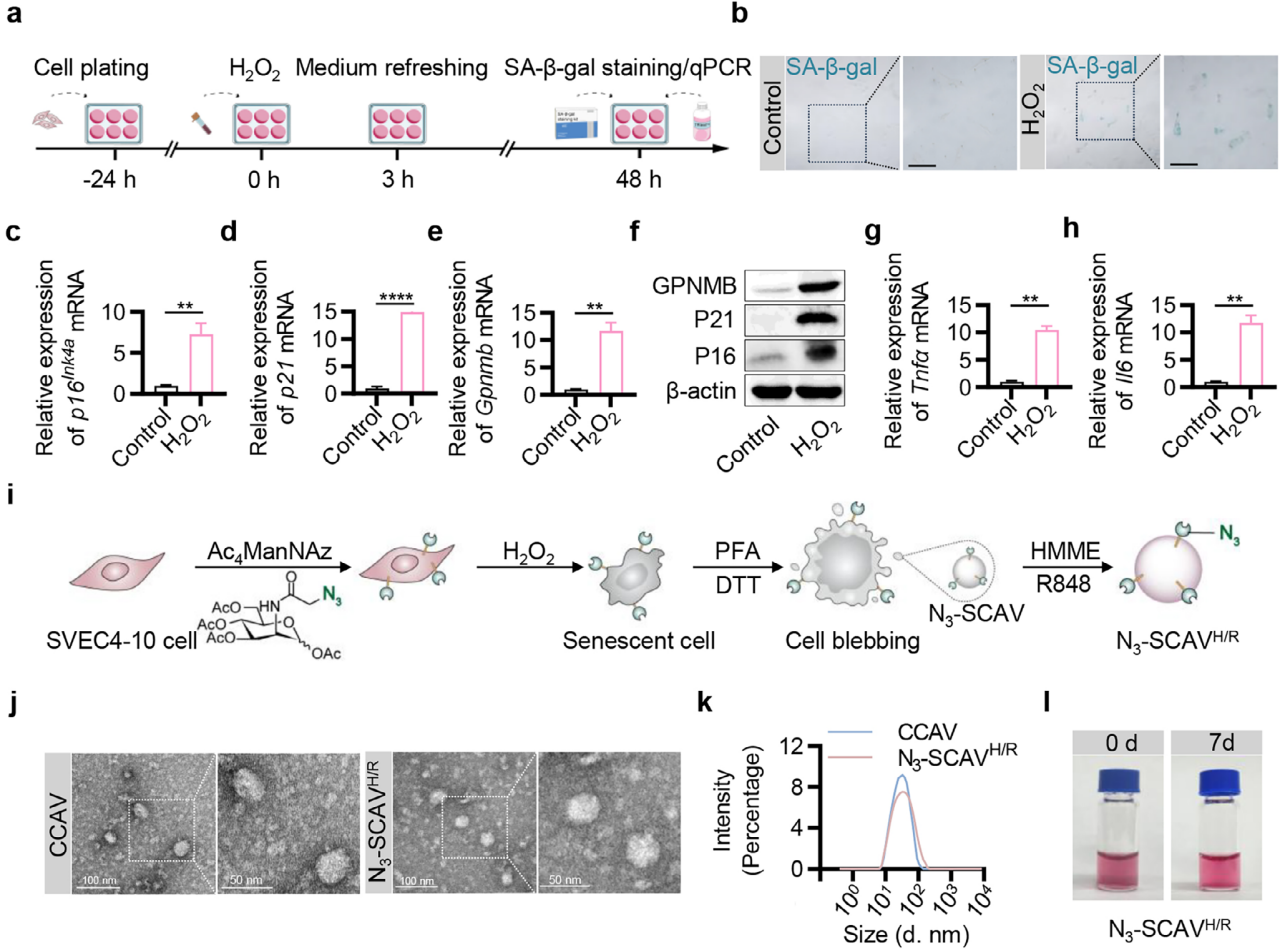
Preparation and characterization of SCAV‐based senolytic sonovaccine. (a) Experimental scheme for inducing cellular senescence. (b) SA‐β‐gal staining of SVEC4‐10 cells after indicated treatments. Scale bar = 100 µm. (c–e) qPCR analysis of (c) *p16^Ink4a^*, (d) *p21*, and (e) *Gpnmb* mRNA levels in H_2_O_2_‐treated and control cells. (f) Western blot analysis of key senescence‐associated markers. (g,h) qPCR analysis of SASP genes (g) *Tnfα* and (h) *Il6*. (i) Schematic illustrating the preparation steps of N_3_‐SCAV^H/R^. (j) Representative TEM image of CCAV or N_3_‐SCAV^H/R^. (k) Representative hydrodynamic size distribution of CCAV or N_3_‐SCAV^H/R^. (l) Representative digital images of N_3_‐SCAV^H/R^ before and after 7 d of storage at 4°C in the dark. Data are expressed as mean ± SEM. Statistical significance was determined by Student's *t*‐test. ^**^ *p* < 0.01, ^****^ *p* < 0.0001.

Previous studies have established that in vitro cellular senescence models express multiple senescence markers, and that block sulfhydryl induced CAVs retain intact membrane surface and intracellular antigenic epitopes. Therefore, SVEC4‐10 cells were subjected to induced senescence to produce senescent antigens (Figure 5a). Compared with the control group, H_2_O_2_‐treated SVEC4‐10 cells exhibited significantly elevated senescence‐associated β‐galactosidase (SA‐β‐gal) activity, along with markedly upregulated expression of senescence markers P16^Ink4a^, P21, and GPNMB (Figure 5b–f). Furthermore, the expression of key senescence‐associated secretory phenotype (SASP) factors, including *Tnfα* and *Il6*, was substantially increased (Figure 5g,h). These results confirm the successful establishment of the senescent cell model.

Azide‐modified senescence‐associated antigen carriers were then prepared via metabolic glycan labeling, H_2_O_2_ treatment, and sulfhydryl blocking, followed by loading with the sonosensitizer HMME and the adjuvant R848 to construct a senolytic sonovaccine (termed N_3_‐SCAV^H/R^), which exhibited encapsulation efficiencies of 36.19% ± 0.62% for HMME and 31.73% ± 3.60% for R848 (Figure 5i). TEM images revealed that N_3_‐SCAV^H/R^ nanoparticles possessed a uniform spherical morphology (Figure 5j). DLS analysis indicated a narrow hydrodynamic size distribution below 100 nm, which showed no significant difference compared to control cell‐derived artificial vesicles (CCAVs) (Figure 5k). The N_3_‐SCAV^H/R^ suspension retained excellent colloidal stability, remaining clear, transparent, and free of precipitate after 7 days of storage at 4°C in the dark; notably, DLS reanalysis confirmed that the hydrodynamic size distribution remained unchanged over this period (Figure 5l; Figure S16). These results indicate that azide modification, H_2_O_2_ induction, and drug loading did not adversely affect the vesicle morphology, hydrodynamic size distribution, or colloidal stability.

Thus, SenoVac is defined as a modular, dual‐component sonovaccine platform consisting of two sequentially administered components: DSPE‐PEG‐DBCO and N_3_‐SCAV^H/R^. The senolytic efficacy of SenoVac was next evaluated using *p16‐tdTomato* reporter mice. Following subcutaneous administration, SenoVac treatment resulted in a notable reduction in senescent cell burden in multiple organs, compared with control groups. Furthermore, the clearance effect was most pronounced in mice that received additional US stimulation (Figure S17). These findings establish SenoVac as an effective senolytic sonovaccine that clears senescent cells in vivo, an effect potentiated by US.

### 2.5 SenoVac Alleviates Atherosclerotic Plaque Development with the Assistance of US

**FIGURE 6:**
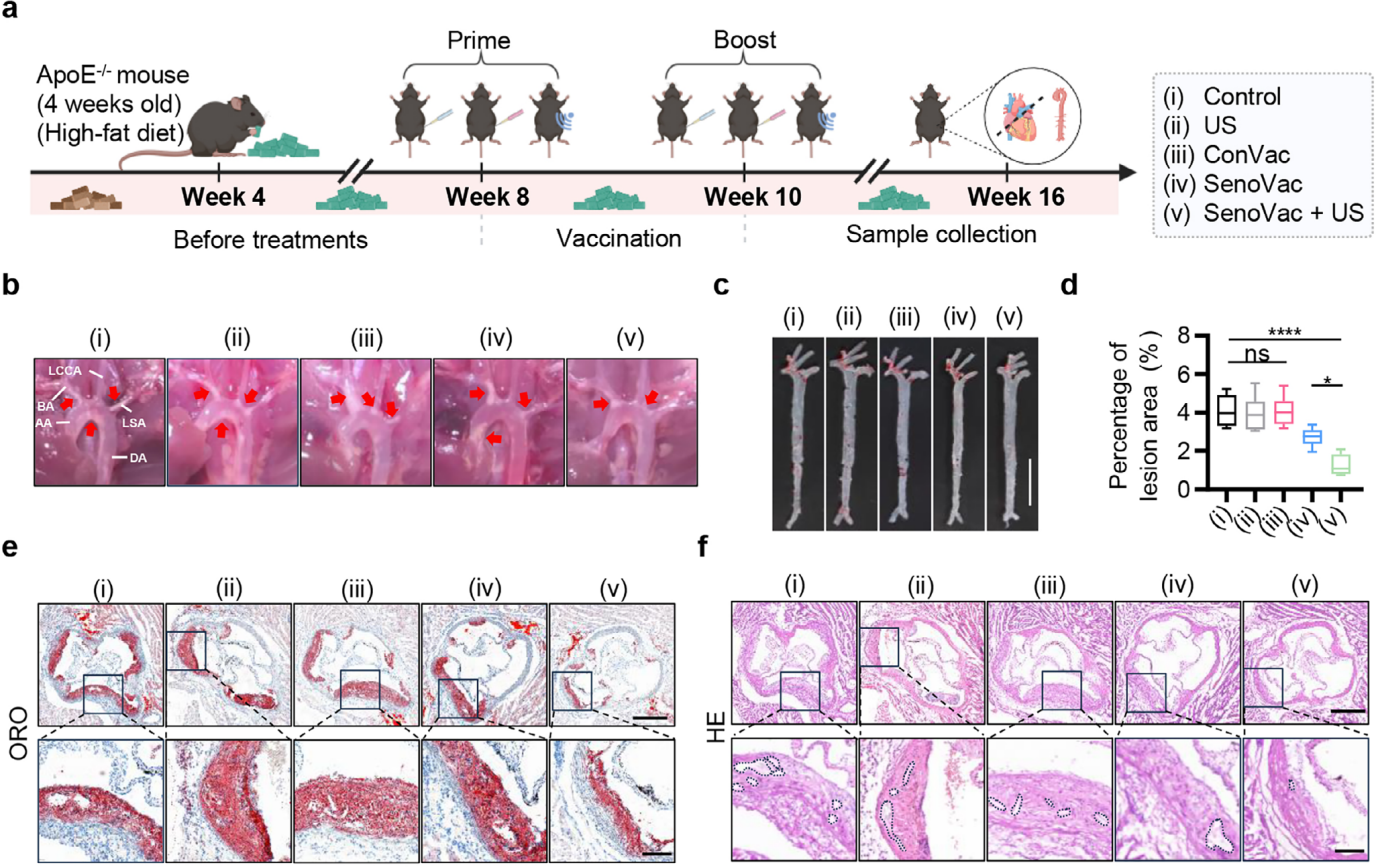
SenoVac alleviates atherosclerotic plaque development with the assistance of US. (a) Schematic of the in vivo experimental design for AS induction in high‐fat diet fed ApoE^−/−^mice. (b) Representative en face views of the aortic arch showing atherosclerotic lesions (red arrows). (c) Representative ORO‐stained whole‐aorta images from ApoE^−/−^ mice under the indicated treatments, Oil Red O stains fat (red). Scale bar = 1 cm. (d) Quantification of the total aortic atherosclerotic lesion area from (c). (e) Representative cross‐sections of the aortic sinus stained with ORO. Scale bar = 400 µm/100 µm. (f) Representative cross‐sections of the aortic sinus stained with H&E. Scale bar = 400 µm/100 µm. AA: ascending aorta; DA: descending aorta; BA: brachiocephalic artery; LCCA: left common carotid artery; LSA: left subclavian artery. Data are expressed as mean ± SEM, n = 6. Statistical significance was determined by one‐way ANOVA with Tukey's post hoc test. ^*^ *p* < 0.05, ^****^ *p* < 0.0001, ns, not significant.

Accumulating evidence, including our current data, indicates that atherosclerotic plaques harbor a substantial burden of senescent cells, suggesting their clearance via a senolytic vaccine may thus represent a promising therapeutic strategy for AS (Figure S18) [32]. To this end, the atheroprotective potential of the SenoVac was assessed in ApoE^−/−^ mice. The mice were fed a high‐fat diet starting at 4 weeks of age. They received a prime vaccination at week 8, a booster at week 10, and were sacrificed for analysis at week 16 (Figure 6a). All mice were pretreated with DSPE‐PEG‐DBCO prior to the administration of either N_3_‐CCAV (termed ConVac) or N_3_‐SCAV^H/R^ (termed SenoVac).

Gross inspection of aortas revealed a modest reduction in atherosclerotic plaque area in mice treated with SenoVac, with a more substantial reduction observed in the SenoVac + US group, compared to controls receiving PBS, US alone, or ConVac (Figure 6b). Consistent with these observations, en face Oil Red O (ORO) staining of the entire aortic tree yielded similar results (Figure 6c,d). In addition, cross‐sectional ORO staining of aortic roots demonstrated a pronounced decrease in atherosclerotic plaque burden, particularly within lipid‐rich regions, in the SenoVac + US group versus all other groups (Figure 6e; Figure S19). Plaque stability was further assessed by measuring necrotic core size in Hematoxylin and Eosin staining (H&E) sections, as an enlarged necrotic core is indicative of higher plaque vulnerability. Histological analysis revealed a significant reduction in the necrotic core area of plaques from the SenoVac + US group, suggesting an improvement in plaque stability (Figure 6f). Functional assessment further revealed that SenoVac treatment significantly reduced aortic pulse wave velocity (PWV), a key indicator of arterial stiffness, with the SenoVac + US group showing the greatest improvement (Figure S20). The observed restoration of vascular compliance provides compelling functional evidence that SenoVac ameliorates AS, with its efficacy being markedly potentiated by US stimulation. These findings establish that SenoVac vaccination effectively attenuates atherosclerotic progression, and that its protective action is substantially enhanced through synergistic combination with US.

### 2.6 Mechanism of SenoVac against AS in ApoE^−/−^ Mice

**FIGURE 7:**
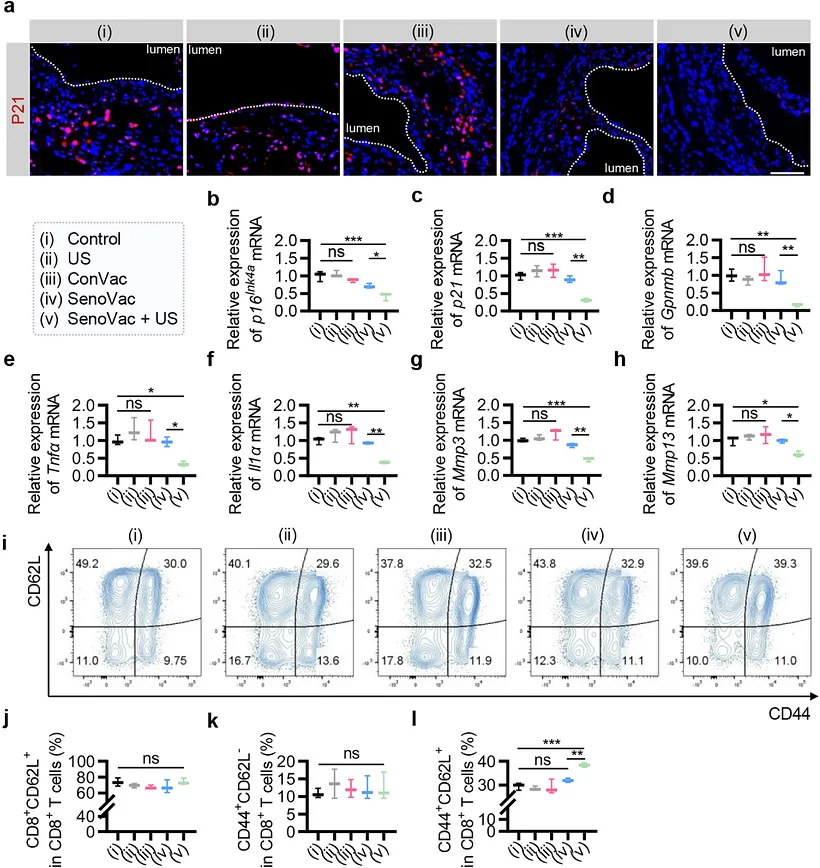
Mechanism of SenoVac against AS in ApoE^−/−^ mice. (a) Immunofluorescence staining of P21 in aortic root sections from ApoE^−/–^ mice under the indicated treatments. Scale bar = 100 µm. (b–d) qPCR analysis of senescence‐related gene expression in aortic roots: (b) *p16^Ink4a^*, (c) *p21*, and (d) *Gpnmb*. (e–h) qPCR analysis of SASP factor mRNA levels in lesioned aortas: (e) *Tnfα*, (f) *Il1α*, (g) *Mmp3*, and (h) *Mmp13*. (i) Representative flow cytometry plots of memory T cell populations in the spleens of ApoE^−/−^ mice under the indicated treatments. (j–l) Quantification of memory T cell subsets from (i): CD8^+^CD62L^+^ T cells (j), CD8^+^ effector memory T cells (CD8^+^CD44^+^CD62L^−^) (k), and CD8^+^ central memory T cells (CD8^+^CD44^+^CD62L^+^) (l). Data are expressed as mean ± SEM. Statistical significance was determined by one‐way ANOVA with Tukey's post hoc test. ^*^ *p* < 0.05, ^**^ *p* < 0.01, ^***^ *p* < 0.001, ns, not significant.

To elucidate the mechanism by which AS is attenuated by SenoVac combined with US, the presence of senescent cells within the plaques was assessed. Immunofluorescence staining of aortic root sections from ApoE^−/−^ mice revealed a significant reduction in P21 positive cells in the SenoVac‐treated group, with a further reduction achieved in the SenoVac + US group (Figure 7a; Figure S21). This demonstrates the efficacy of the combined treatment in clearing senescent cells from atherosclerotic lesions. Consistent with this, qPCR analysis of aortic tissues showed that the combined SenoVac + US treatment considerably suppressed the expression of senescence markers (*p16^Ink4a^*, *p21*, *Gpnmb*) and major SASP factors (*Tnfα*, *Il1α*, *Mmp3*, *Mmp13*) compared to SenoVac alone or control groups (Figure 7b–h). These results indicate that the synergy of SenoVac and US stimulation effectively reduces the burden of senescent cells and dampens the SASP within aortic plaques, uncovering a key mechanism through which this strategy mitigates the progression of AS.

The systemic immune status in ApoE^−/−^ mice was next assessed at the 16‐week endpoint. Splenocytes were isolated and analyzed by flow cytometry. While no significant differences were observed in the overall populations of CD3^+^CD4^+^ and CD3^+^CD8^+^ effector T cells across groups, this finding is consistent with two plausible explanations: the potent systemic proinflammatory milieu induced by the high‐cholesterol diet may have masked vaccine‐specific T cell responses, and/or the extended interval (6 weeks) since the last immunization may have allowed effector T cell numbers to return to baseline (Figure S22). We therefore focused on the establishment of immunological memory, a more durable indicator of vaccine efficacy. Although the proportion of CD8^+^ effector memory T cells (Tem, CD8^+^CD44^+^CD62L^−^) was comparable among groups, mice treated with SenoVac combined with US exhibited a significant increase in CD8^+^ central memory T cells (Tcm, CD8^+^CD44^+^CD62L^+^) (Figure 7i–l). This result suggests that the combined treatment promoted the formation of a pool of long‐lived memory T cells, which may be capable of rapid recall upon antigen re‐encounter; however, functional validation, such as re‐excitation or adoptive transfer studies, is required to confirm their capacity to mount effective secondary responses against target senescent cells.

### 2.7 Protective Effects on Multi‐Organs and Biosafety of SenoVac

**FIGURE 8:**
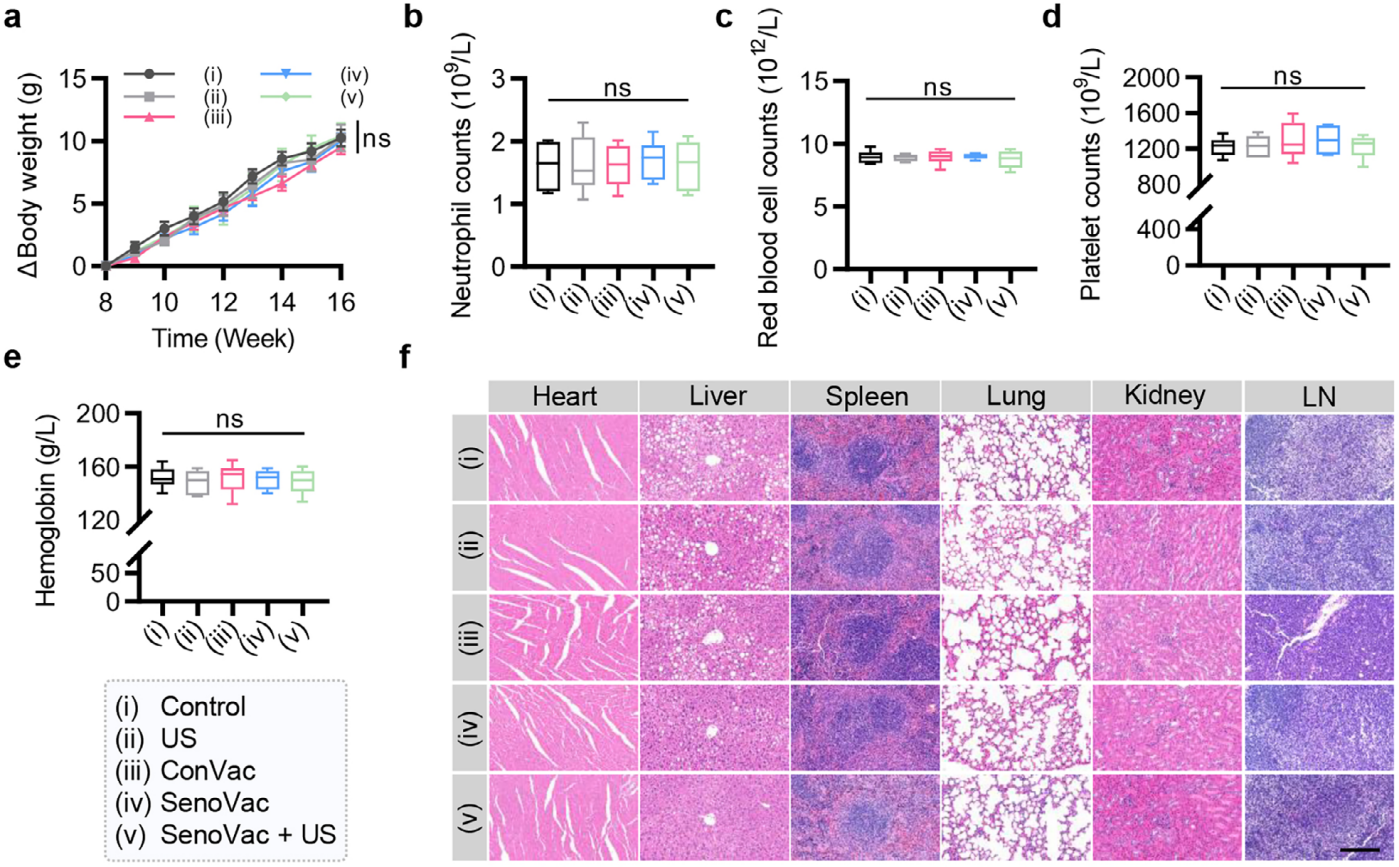
Good biosafety of SenoVac. (a) Body weight changes over time in ApoE^−/−^ mice under the indicated treatments. (b) Neutrophil counts. (c) Red blood cell counts. (d) Platelet counts. (e) Hemoglobin levels. (f) Histopathological analysis of major organs (heart, liver, spleen, lung, kidney), and dLNs from treated ApoE^−/−^ mice. Scale bar = 200 µm. Data are expressed as mean ± SEM, n = 6. Statistical significance was determined by two‐way ANOVA with Tukey's post hoc test for (a), and by one‐way ANOVA with Tukey's post hoc test for (b‐e). ns, not significant.

Identical mouse models and dosing protocols were used to ensure consistency in the therapeutic trials in vivo. The biosafety of the treatment was evaluated through continuous monitoring of mouse body weight after prime vaccination (weeks 8–16), which showed a comparable gradual increase across all groups, indicating no adverse effects of SenoVac vaccination or US stimulation on normal growth (Figure 8a). Given the frequent association of senolytic agents with hematological toxicities, complete blood count analysis was performed. No significant alterations were observed in neutrophil, red blood cell, or platelet counts, nor in hemoglobin levels, suggesting that SenoVac does not adversely affect major hematopoietic lineages (Figure 8b–e). Blood biochemical analysis was conducted to assess organ function. For renal function, levels of uric acid (UA), creatinine (CREA), and blood urea nitrogen (BUN) did not differ significantly among the groups, suggesting no detectable adverse effect on kidney function (Figure S23). Levels of alanine aminotransferase (ALT), aspartate aminotransferase (AST), and total bilirubin (TBIL) were significantly reduced in SenoVac + US group, while albumin (ALB) levels remained unchanged, suggesting a hepatoprotective effect (Figure S24). US examination demonstrated a significantly lower liver‐to‐kidney ratio in the SenoVac + US group compared to controls and the SenoVac‐alone group (Figure S25). H&E staining showed a marked reduction in hepatic lipid vacuoles in the SenoVac + US group, consistent with ultrasonographic findings (Figure 8f). Finally, echocardiography confirmed the absence of significant effects on cardiac systolic or diastolic function across all groups (Figure S26). In contrast, key blood lipid parameters showed no significant differences (Figure S27). Collectively, these data suggest that SenoVac mitigates the pathology in multi‐organs through clearance of senescent cells. In summary, these comprehensive biosafety data underscore the favorable biosafety of SenoVac vaccination.

## 3 Discussion

In this study, we developed a modular senolytic sonovaccine for targeted LN delivery and US‐enhanced cross‐presentation. The platform employs a two‐step strategy involving pre‐installation of bioorthogonal docking sites in LNs, followed by vaccine accumulation via click chemistry. Focal US then triggers the sonosensitizer to produce ROS, disrupting DC endosomes to promote antigen cross‐presentation. In ApoE^−/−^ mice with AS, SenoVac cleared plaque senescent cells, ameliorated disease, and showed a favorable safety profile.

The rising global burden of age‐related diseases, such as AS, underscores the need for interventions targeting fundamental aging mechanisms [33, 34, 35]. Cellular senescence, driven by permanent cell‐cycle arrest and a pro‐inflammatory secretory phenotype (SASP), is a key contributor to tissue dysfunction and disease progression [15, 36]. Our findings reinforce the premise that eliminating senescent cells can ameliorate or even reverse the progression of age‐related pathologies such as AS.

Both pharmacological senolytics and senolytic vaccines aim to selectively clear senescent cells [37, 38]. While small‐molecule senolytics validate this concept preclinically, their clinical translation is often hampered by dose‐limiting, on‐target/off‐tissue toxicities, such as myelosuppression [39, 40, 41]. Senolytic vaccines pursue the same goal through a more precise, immune‐engaging strategy [4, 5, 6]. By directing adaptive immunity against senescence‐associated antigens, they offer the potential for high specificity, durable protection, and long‐term immune surveillance, minimizing off‐target damage [4, 5, 6].

Rational antigen selection is critical [42]. Potential targets are found within the senescence‐associated surfaceome, including proteins like CD153 [7], GPNMB [8], uPAR [9, 10], DPP4 [11], β‐2 M [12], and NKG2D [13], among others. However, these endogenous antigens are typically weakly immunogenic, and the pronounced heterogeneity of senescent cells renders monovalent approaches inherently limited [6]. We therefore adopted a whole‐cell‐based strategy using SCAVs. Generated from in vitro‐induced senescent cells, SCAVs present a broad, native antigenic repertoire encompassing membrane, cytosolic, and potentially post‐translationally modified proteins. This provides a diverse antigenic payload capable of targeting heterogeneous senescent populations, offering a practical solution that circumvents the need for prior antigen identification. Notably, such carriers may possess multi‐antigenic potential, a possibility that could be further explored through comprehensive proteomic analyses in future studies.

Vaccine efficacy is critically dependent on efficient antigen delivery to and retention within LNs [22]. Although passive lymphatic drainage favors particles sized 10–100 nm, such drainage alone often results in rapid efflux and limited APC uptake [23, 24, 25]. Conventional active targeting by direct ligand conjugation creates a design conflict: such modifications often increase hydrodynamic size, impairing passive drainage, while attempts to compensate by reducing core particle size inevitably sacrifice antigen payload [22]. To resolve this, we implemented a decoupled “two‐step” strategy. A small targeting moiety first installs docking sites in LNs, after which optimally sized vaccine nanoparticles accumulate via bioorthogonal ligation. This approach preserves the ideal hydrodynamic diameter of each component while enabling active retention, a modular strategy applicable to diverse nanovaccine platforms. Although catalyst‐free click chemistry (SPAAC) was chosen for its fast kinetics and biocompatibility, the core targeting concept can be adapted to other high‐affinity binding systems [43, 44, 45].

The induction of robust CTL responses by protein‐based vaccines relies on efficient cross‐presentation, a process limited by poor endo‐lysosomal antigen escape [19, 20, 28, 29]. Current enhancement strategies include cationic polymers (e.g., exploiting the proton‐sponge effect) [46], pH‐sensitive membrane‐destabilizing materials [47, 48], and light‐activated photosensitizers that generate ROS [49]. ROS‐mediated approaches offer a dual advantage by permeabilizing endosomal membranes and potentially enhancing subsequent proteasomal processing [50, 51, 52, 53]. We adopted a sonodynamic strategy by incorporating the sonosensitizer HMME into the vaccine nanoparticles. Compared to light, US provides superior tissue penetration and spatiotemporal control, enabling non‐invasive activation without the phototoxicity associated with optical methods [54]. This strategy robustly enhanced antigen‐specific CD8^+^ T cell responses in our models while maintaining high biocompatibility. From a translational perspective, US stimulation can be applied directly to the vaccination‐site LN, decoupling the need to target deep tissues such as atherosclerotic plaques. Optimizing US parameters to achieve endosomal escape without inducing off‐target thermal effects or significant cell death will be a critical step prior to clinical translation, particularly given anatomical differences between small animals and humans.

Several limitations merit consideration. First, the antigenic profile of the in vitro‐induced senescent cell model, while broad, may not fully recapitulate the heterogeneity of senescent cells in vivo, particularly across different tissues and disease contexts. Second, while the TLR7/8 agonist R848 used in our study induced only transient elevations in TNF‐α and IL‐6, with no notable local inflammation observed in LN histology, systemic inflammatory responses remain a known concern for potent adjuvants. From a preventive vaccination perspective, administration prior to plaque formation may mitigate this risk. Nonetheless, the risk–benefit balance of employing a potent TLR agonist in the context of a chronic inflammatory condition such as AS warrants careful consideration. Third, the long‐term durability of the immune response and the potential for immune tolerance or exhaustion with repeated vaccination remain to be fully investigated. Finally, while our murine model of AS demonstrated efficacy and safety, translational studies in larger animal models are necessary to further evaluate the platform's clinical potential.

In summary, we have developed and validated a modular senolytic sonovaccine platform that synergistically integrates whole‐cell vesicles with diverse antigenic payloads, active LN targeting via bioorthogonal chemistry, and US‐enhanced cross‐presentation. This integrated strategy effectively addresses key bottlenecks in senolytic vaccine development, namely broad antigen coverage, efficient LN delivery and retention, and potent CD8^+^ T cell priming. Our work establishes a promising and translatable framework for the immunotherapy of senescence‐associated diseases.

## 4 Conclusions

In this study, we have developed SenoVac, a modular dual‐component sonovaccine platform that integrates active LN targeting and stimulus‐responsive cross‐presentation enhancement to address key challenges in senolytic immunotherapy. This design enables efficient delivery and retention of a broad senescent antigen repertoire within LNs, where US‐triggered ROS generation promotes endosomal escape and robust CD8^+^ T cell activation. In an ApoE^−/−^ mouse model of AS, SenoVac demonstrated remarkable efficacy and a favorable safety profile, with no detectable off‐target toxicity to healthy tissues. Our work represents a paradigm shift in senolytic immunotherapy by addressing longstanding limitations of current strategies in a single integrated platform. By establishing a translatable strategy that unites these innovations, we lay the groundwork for next‐generation senolytic vaccines not only for AS but also for other age‐related diseases driven by cellular senescence.

## 5 Materials and Methods

### 5.1 Materials

All chemical reagents and antibodies were procured from commercial suppliers. Specifically, DSPE‐PEG‐DBCO (HY‐W440835), Ac4ManNAz (HY‐118297), R848 (HY‐13740), and HMME (HY‐134990) were obtained from MedChemExpress (Monmouth Junction, NJ, USA). The Lyso‐Tracker Green probe (C1047S) and senescence β‐galactosidase staining kit (C0602) were sourced from Beyotime Biotechnology Co., Ltd. (Shanghai, China). DCFH‐DA (GC30006) was obtained from GlpBio Technology (Montclair, CA, USA). Flow cytometry reagents, including anti‐mouse H‐2Kb bound to SIINFEKL‐PE (141604), CD11c‐APC (117310), CD86‐PE (105008), CD80‐FITC (104706), CD45‐PerCP/Cyanine5.5 (103130), CD3‐FITC (100204), CD4‐APC (100412), CD8a‐PE (100708), CD44‐BV650 (103049), CD62L‐BV605 (104438), Zombie Aqua Fixable Viability Kit (423101), TruStain FcX (101320), cell staining buffer (420201), and RBC lysis buffer (420302), were purchased from BioLegend, Inc. (San Diego, CA, USA).

### 5.2 Cell Culture

The murine endothelial cell line SVEC4‐10, along with HEK293T and DC2.4 cell lines, was procured from the China Center for Type Culture Collection (CCTCC, Wuhan, China). Cells were cultured at 37°C in a humidified 5% CO_2_ incubator. The culture media used were as follows: DMEM for SVEC4‐10 and HEK293T cells, and RPMI 1640 for DC2.4 cells. All media formulations contained 10% fetal bovine serum (FBS) and 1% penicillin‐streptomycin.

### 5.3 CAVs Preparation, Purification, and Drugs Loading

Donor cells were pelleted from culture medium at 200 g for 5 min and resuspended in Dulbecco's PBS (10^7^ cells mL^−1^) with 25 mm PFA (Thermo Fisher Scientific) and 2 mm DTT (Thermo Fisher Scientific). After 2 h of incubation at 37 °C, CAVs were isolated by sequential centrifugation: first at 200 g for 5 min to remove cells, then at 9300 g for 10 min to pellet debris and microvesicles. The supernatant was concentrated using a 30 kDa centrifugal filter (3200 g, 15 min) and washed twice with PBS. CAVs protein concentration was determined by BCA assay.

CAVs were incubated with HMME and R848 for 12 h at 4°C in the dark. Following incubation, the solution was concentrated using a 30‐kDa centrifugal filter (3200 g, 15 min). The retained CAV^H/R^ were washed three times with PBS using centrifugal filtration and resuspended in PBS. The final product was stored at 4°C in the dark.

### 5.4 In Vitro Binding Visualization of the Modular LN‐Targeted Aggregation System

DSPE‐PEG or DSPE‐PEG‐DBCO was mixed with bovine serum albumin (BSA) at a 1:5 molar ratio and incubated overnight to form albumin‐DSPE‐PEG or albumin‐DSPE‐PEG‐DBCO complexes. To evaluate the interaction between N_3_‐CAVs and DBCO‐functionalized cell surfaces, SVEC4‐10 cells were incubated with the albumin‐DSPE‐PEG or albumin‐DSPE‐PEG‐DBCO complexes for 24 h. After washing three times to remove unbound complexes, the cells were treated with DiI‐labeled N_3_‐CAVs for 5 min, washed again to remove nonspecifically bound vesicles, and imaged using a confocal laser scanning microscope.

### 5.5 Lymphatic Drainage Efficiency of the Modular LN‐Targeted Aggregation System

To assess the lymphatic drainage efficiency of the system, male C57BL/6 mice (6‐8‐week‐old) received subcutaneous injections of DSPE‐PEG‐DBCO or DSPE‐PEG (60 nmol per mouse) into the left inguinal region. After 24 h, DiR‐labeled N_3_‐CAVs were administered via subcutaneous injection into the left footpad. Fluorescence intensity in the inguinal LNs was evaluated 24 h later using in vivo and ex vivo IVIS imaging, followed by quantitative analysis of lymphatic drainage efficiency.

### 5.6 Biodistribution of the Modular LN‐Targeted Aggregation System

Male C57BL/6 mice (6‐8 weeks old) were subcutaneously injected with DSPE‐PEG‐FITC (60 nmol per mouse; n = 3 per group). Inguinal LNs were collected at 12, 24, 48, 72, and 120 h post‐injection and imaged using an IVIS Spectrum imaging system (PerkinElmer, Waltham, MA, USA).

To further evaluate the in vivo biodistribution of the system, mice were injected subcutaneously in the left inguinal region with DSPE‐PEG‐DBCO or DSPE‐PEG (60 nmol per mouse). After 24 h, DiR/DiO‐labeled N_3_‐CAVs were administered via subcutaneous injection. Mice were euthanized 24 h later, and major organs (heart, liver, spleen, lung, kidney), along with dLNs were collected. Biodistribution was analyzed at both macroscopic and histological levels using in vivo fluorescence imaging and confocal microscopy of tissue sections.

### 5.7 Plasmid Construction

An OVA fragment was synthesized and cloned into the pcDNA3.1(‐) plasmid by GenScript Biotech (Nanjing, China). All gene sequences used for synthesis are provided in Table S1.

### 5.8 Transfection

HEK293T cells were transfected with 10 µg of the respective plasmids using Lipofectamine 2000 (Invitrogen, USA) according to the manufacturer's instructions. After 6 h, the medium was replaced with complete culture medium containing 10% FBS. Cells were further cultured for 48 h, after which they were harvested for the preparation of CAV^OVA^.

### 5.9 Cellular Uptake of CAVs In Vitro

DC2.4 cells were plated on glass‐bottom confocal dishes and incubated with DiI‐labeled CAVs for 12 h. After washing to remove non‐internalized CAVs, the cells were fixed with 4% paraformaldehyde for 10 min and gently washed three times with PBS. Nuclei were then stained with Hoechst 33342. Fluorescent images were captured using a confocal laser scanning microscope (Nikon A1R HD25, Nikon Instruments Inc., Tokyo, Japan).

### 5.10 Detection of Intracellular ROS in DCs

DCs were plated in glass‐bottom confocal dishes and incubated with CAV^Ctrl^ or CAV^OVA/H^ for 12 h. After removal of non‐internalized CAVs by washing, cells were stained with 10 µm DCFH‐DA for 30 min at 37 °C. Immediately following another wash, selected groups were subjected to US stimulation (1 W cm^−^^2^, 1 min; HANIL TM HS‐501). The cells were then washed, counterstained with Hoechst 33342 for nuclei visualization, and analyzed by CLSM to assess ROS production.

### 5.11 Analysis of DCs Maturation and Antigen Presentation In Vitro

DCs were treated with the indicated CAVs for 12 h, washed three times with PBS, and replenished with fresh medium. Selected groups were then exposed to US irradiation (1 W cm^−^^2^, 1 min). For maturation analysis, cells were further cultured for 48 h, blocked with TruStain FcX, and stained with anti‐mouse CD86‐PE and anti‐mouse CD80‐FITC antibodies for 20 min prior to analysis by BD FACSCelesta flow cytometer (BD Biosciences, San Jose, CA, USA). To evaluate antigen presentation, cells were blocked with TruStain FcX and stained with anti‐mouse H‐2Kb bound to SIINFEKL‐PE antibody for 20 min, followed by flow cytometry. Data from all experiments were analyzed using FlowJo software (v10.8.1).

### 5.12 Senescence Induction

To induce cellular senescence, SVEC4‐10 cells (under 10 passages) were treated with 300 µm H_2_O_2_ for 3 h. Following this treatment, the cells were washed and maintained in fresh culture medium for an additional 48 h prior to subsequent experiments.

### 5.13 SA‐β‐gal Assay

SVEC4‐10 cells were seeded in 12‐well plates at a density of 1×10^5^ cells per well and subjected to senescence induction by H_2_O_2_ treatment. SA‐β‐gal activity was assessed using a commercial senescence β‐galactosidase staining kit. Images were acquired with an inverted fluorescence microscope (model IX73, Olympus Corporation, Tokyo, Japan).

### 5.14 Preparation of SCAV‐Based Senolytic Sonovaccine

SVEC4‐10 cells were seeded in 100 mm dishes and cultured in complete medium containing 50 µm Ac_4_ManNAz until they reached 80%–90% confluency. Cellular senescence was then induced by exposure to 300 µm H_2_O_2_ for 3 h. After induction, the cells were washed and maintained in fresh medium containing 50 µm Ac_4_ManNAz for 2 days. The resulting senescent cells were harvested and resuspended in Dulbecco's PBS containing 25 mm PFA and 2 mm DTT to stimulate vesiculation. N_3_‐functionalized SCAVs (N_3_‐SCAVs) were isolated and purified from the supernatant, quantified by BCA assay, and subsequently co‐incubated with HMME and R848 to form the final N_3_‐SCAV^H/R^ construct.

### 5.15 RNA Isolation and Quantitative Real‐Time PCR

Total RNA was extracted from tissues, cultured cells, or CAVs using Tripure Isolation Reagent according to the manufacturer's instructions. cDNA was synthesized from mRNA using a First Strand cDNA Synthesis Kit (Genenode, Beijing, China). Quantitative PCR was performed with FastStart Essential DNA Green Master (Roche, Indianapolis, IN, USA) and gene‐specific primers (sequences listed in Table S2). The expression of target genes was normalized to a reference gene, and relative expression levels were calculated using the 2–^ΔΔCT^ method.

### 5.16 Western Blot Analysis

Protein samples of equal quantity were separated by 12% SDS‐PAGE and subsequently transferred onto a nitrocellulose membrane in an ice bath. The nitrocellulose membranes were blocked with 5% BSA for 1 h at room temperature, followed by incubation with primary antibodies against P16^INK4A^, P21, GPNMB, and β‐actin overnight at 4°C. After three washes with Tris‐buffered saline containing 0.1% Tween 20 (TBS‐T), the membranes were incubated with appropriate horseradish peroxidase (HRP)‐conjugated secondary antibodies for 1 h at room temperature. Protein bands were visualized using an Omni‐ECL Femto Light Chemiluminescence Kit (Epizyme Biomedical Technology, Shanghai, China) and imaged with a ChemiDoc MP imaging system (Bio‐Rad Laboratories, Hercules, CA, USA).

### 5.17 Experimental Animals

6‐8‐week‐old male C57BL/6 mice were purchased from the Model Animal Research Center of the Fourth Military Medical University. Male ApoE^−/−^ mice (C57BL/6 background) were obtained from GemPharmatech Co., Ltd. (Nanjing, China) at 4 weeks of age. Mice were fed a high‐fat diet (D12492, Research Diets; 45% kcal from fat, 20% kcal from protein, 35% kcal from carbohydrate) from 4 to 16 weeks of age. Within this period, mice received a primary vaccination at 8 weeks and a booster at 10 weeks. All mice were sacrificed for analysis at 16 weeks of age.

### 5.18 Histological Analysis

Following anesthesia and transcardial perfusion, experimental mice were euthanized. Major organs, including the aorta, heart, liver, spleen, lung, kidney, and dLNs were collected and carefully dissected to remove surrounding adipose tissue. Tissues were fixed in 4% PFA for 24 h at 4°C. Fixed specimens were subsequently cryoprotected in 30% sucrose solution overnight at 4°C, then embedded in optimal cutting temperature (OCT) compound and sectioned at 5 µm thickness using a cryostat. Serial sections were stained with ORO and H&E according to established protocols. Histopathological evaluation of H&E‐stained sections from all major organs was performed independently by two board‐certified pathologists. Lesion size and lipid core area in aortic sections were quantified using ImageJ software (National Institutes of Health, Bethesda, MD, USA).

### 5.19 Flow Cytometric Analysis of Immune Activation Profiles In Vivo

C57BL/6 mice: at 3 days and 10 days post‐treatment, immunized C57BL/6 mice were anesthetized and dLNs were harvested. Tissues were mechanically dissociated and passed through a 70‐µm cell strainer to generate single‐cell suspensions. After centrifugation at 1800 rpm for 10 min at 4°C, cells were stained with Zombie Aqua Fixable Viability Kit and blocked with TruStain FcX. For DC maturation analysis, cells were stained with anti‐mouse CD11c‐APC, CD86‐PE, and CD80‐FITC antibodies for 20 min. Antigen presentation was evaluated using anti‐mouse CD11c‐APC and anti‐mouse H‐2Kb bound to SIINFEKL‐PE antibody. T cell activation was assessed by staining with anti‐mouse CD45‐PerCP/Cyanine5.5, CD3‐FITC, and CD8a‐PE antibodies. All samples were analyzed on flow cytometer.

ApoE^−/−^ mice: Spleens were collected from ApoE^−/–^ mice at 16 weeks of age. Single‐cell suspensions were prepared by mechanical dissociation and erythrocyte lysis, followed by filtration through a 70‐µm strainer. Then, cells were stained with Zombie Aqua Fixable Viability Kit and blocked with TruStain FcX. To assess immunological memory formation, cells were stained with anti‐mouse CD45‐PerCP/Cyanine5.5, CD3‐FITC, CD4‐APC, CD8a‐PE, CD44‐BV650, and CD62L‐BV605 antibodies for 20 min. Data from all experiments were analyzed using FlowJo software (v10.8.1).

### 5.20 Serum ELISA, Serum Biochemistry, and Cholesterol Level Analysis

Blood samples were collected from C57BL/6 mice via retro‐orbital bleeding at 3 d and 10 d post‐treatment. Serum was separated by centrifugation and stored at −80°C until analysis. Cytokine concentrations (IL‐6, TNF‐α, and IFN‐γ) were quantified using commercial ELISA kits (MultiSciences, Hangzhou, China) according to the manufacturer's instructions.

For ApoE^−/−^ mice, blood was obtained at 16 weeks of age following an 8‐h fasting period. Renal function was evaluated by measuring UA, CREA, and BUN levels. Hepatic function was assessed through aspartate AST, ALT, ALB, and TBIL measurements. Lipid profiles were determined by quantifying TC, HDL‐C, LDL‐C, and TG levels.

### 5.21 Statistical Analysis

All data are expressed as mean ± SEM. Statistical comparisons between two groups were performed using Student's t‐test. For comparisons involving multiple groups, one‐way ANOVA with Tukey's post hoc test was applied. Body weight changes over time were analyzed using two‐way ANOVA with Tukey's post hoc test. Statistical analyses were conducted using GraphPad Prism 9.5, with ^*^ *p* < 0.05 considered statistically significant (^*^ *p* < 0.05, ^**^ *p* < 0.01, ^***^ *p* < 0.001, ^****^ *p* < 0.0001).

### 5.22 Ethical Statement

All animal experiments were performed in accordance with the National Institutes of Health Guide for the Care and Use of Laboratory Animals and were approved by the Institutional Animal Care and Use Committee of The Fourth Military Medical University (Approval No. 20250153).

## Author Contributions

**Lijun Yuan**, **Xuekang Yang**, and **Guodong Yang**: conceptualization, funding acquisition; **Liang Zhang**, **Yubo Lai**, **Jia Wang**, **Lingling Liu**, **Jieyuan An**, **Mi Qu**, **Yuan Liang**, and **Bijun Tan**: investigation. **Liang Zhang**, **Yubo Lai**, **Jia Wang**, and **Lingling Liu**: methodology; **Lijun Yuan**, **Xuekang Yang**, and **Guodong Yang**: supervision; **Liang Zhang**, **Yubo Lai**, and **Jia Wang**: writing – original draft; **Lijun Yuan**, **Xuekang Yang**, and **Guodong Yang**: writing – review & editing.

## Conflicts of Interest

The authors declare no conflicts of interest.

## Supporting information




**Supporting File**: advs75770‐sup‐0001‐SuppMat.docx.

## Data Availability

The data that support the findings of this study are available from the corresponding author upon reasonable request.
