# Supplementary material for: Senescent Cell Derived Artificial Vesicle‐Based Senolytic Sonovaccine Platform with Augmented Lymph Node Delivery and Antigen Cross‐Presentation Efficacy
Source: Adv Sci (Weinh). 2026 May 20;13(46):e75770. doi: 10.1002/advs.75770 (PMC13335959; doi:10.1002/advs.75770)
Supplement: Supplementary file 1 [file ADVS-13-e75770-s001.docx]

Supporting Information

**Senescent Cell Derived Artificial Vesicle-Based Senolytic Sonovaccine Platform with Augmented Lymph Node Delivery and Antigen Cross-Presentation Efficacy**

Liang Zhang^1^ | Yubo Lai^1^ | Jia Wang^1^ | Lingling Liu^2^ | Jieyuan An^1^ | Mi Qu^1^ | Yuan Liang^1^ | Bijun Tan^1^ | Guodong Yang^3^ | Xuekang Yang^2^ | Lijun Yuan^1^

^1^Department of Ultrasound Medicine, Tangdu Hospital, The Fourth Military Medical University, Xi’an, Shaanxi, 710038, China | ^2^Department of Burns and Cutaneous Surgery, Xijing Hospital, The Fourth Military Medical University, Xi’an, Shaanxi, 710032, China | ^3^State Key Laboratory of Holistic Integrative Management of Gastrointestinal Cancers, Department of Biochemistry and Molecular Biology, The Fourth Military Medical University, Xi’an, Shaanxi, 710032, China

**Correspondence:** Lijun Yuan ([yuanlj@fmmu.edu.cn](mailto:yuanlj@fmmu.edu.cn)) | Xuekang Yang ([yangxuekangburns@163.com](mailto:yangxuekangburns@163.com)) | Guodong Yang ([yanggd@fmmu.edu.cn](mailto:yanggd@fmmu.edu.cn))

Liang Zhang, Yubo Lai, and Jia Wang contributed equally to this work.

**Table S1 | Sequences of the plasmids used in this study.**

| **Plasmid** | **Sequence** |
| --- | --- |
| OVA | GGGCTAGCGCCACCATGGGCTCCATCGGCGCAGCAAGCATGGAATTTTGTTTTGATGTATTCAAGGAGCTCAAAGTCCACCATGCCAATGAGAACATCTTCTACTGCCCCATTGCCATCATGTCAGCTCTAGCCATGGTATACCTGGGTGCAAAAGACAGCACCAGGACACAGATAAATAAGGTTGTTCGCTTTGATAAACTTCCAGGATTCGGAGACAGTATTGAAGCTCAGTGTGGCACATCTGTAAACGTTCACTCTTCACTTAGAGACATCCTCAACCAAATCACCAAACCAAATGATGTTTATTCGTTCAGCCTTGCCAGTAGACTTTATGCTGAAGAGAGATACCCAATCCTGCCAGAATACTTGCAGTGTGTGAAGGAACTGTATAGAGGAGGCTTGGAACCTATCAACTTTCAAACAGCTGCAGATCAAGCCAGAGAGCTCATCAATTCCTGGGTAGAAAGTCAGACAAATGGAATTATCAGAAATGTCCTTCAGCCAAGCTCCGTGGATTCTCAAACTGCAATGGTTCTGGTTAATGCCATTGTCTTCAAAGGACTGTGGGAGAAAGCATTTAAGGATGAAGACACACAAGCAATGCCTTTCAGAGTGACTGAGCAAGAAAGCAAACCTGTGCAGATGATGTACCAGATTGGTTTATTTAGAGTGGCATCAATGGCTTCTGAGAAAATGAAGATCCTGGAGCTTCCATTTGCCAGTGGGACAATGAGCATGTTGGTGCTGTTGCCTGATGAAGTCTCAGGCCTTGAGCAGCTTGAGAGTATAATCAACTTTGAAAAACTGACTGAATGGACCAGTTCTAATGTTATGGAAGAGAGGAAGATCAAAGTGTACTTACCTCGCATGAAGATGGAGGAAAAATACAACCTCACATCTGTCTTAATGGCTATGGGCATTACTGACGTGTTTAGCTCTTCAGCCAATCTGTCTGGCATCTCCTCAGCAGAGAGCCTGAAGATATCTCAAGCTGTCCATGCAGCACATGCAGAAATCAATGAAGCAGGCAGAGAGGTGGTAGGGTCAGCAGAGGCTGGAGTGGATGCTGCAAGCGTCTCTGAAGAATTTAGGGCTGACCATCCATTCCTCTTCTGTATCAAGCACATCGCAACCAACGCCGTTCTCTTCTTTGGCAGATGTGTTTCCCCTTAAGAATTCGG |

**Table S2 | Primers used in the study.**

| **Primer** | **Forward** | **Reverse** |
| --- | --- | --- |
| mouse *Cdkn2a* (p16^Ink4a^) | 5’-CGCAGGTTCTTGGTCACTGT-3’ | 5’-TGTTCACGAAAGCCAGAGCG-3’ |
| mouse *Cdkn1a* (p21) | 5’-CCTGGTGATGTCCGACCTG-3’ | 5’-CCATGAGCGCATCGCAATC-3’ |
| mouse *Gpnmb* | 5′-ACGGCAGGTGGAAGGACT-3′, | 5′-CGGTGAGTCACTGGTCAGG-3′ |
| *mouse Psmb8* | 5’-CATTCCTGAGGTCCTTTGGTGGTG-3’ | 5’-ACTTGAAGGCGAGTGTGGTTGTG-3’ |
| *mouse Tap1* | 5’-GGACTTGCCTTGTTCCGAGAG-3’ | 5’-GCTGCCACATAACTGATAGCGA-3’ |
| mouse *Tnfα* | 5’-CTGAACTTCGGGGTGATCGG-3’ | 5’-GGCTTGTCACTCGAATTTTGAGA-3’ |
| mouse *Il6* | 5’-TTCCATCCAGTTGCCTTCTT-3’ | 5’-CAGAATTGCCATTGCACAAC-3’ |
| mouse *Il1α* | 5’-TCCATAACCCATGATCTGGAA-3’ | 5’-TTGGTTGAGGGAATCATTCAT-3’ |
| mouse *Mmp3* | 5’-CAAAACATATTTCTTTGTAGAGGACAA-3’ | 5’-TTCAGCTATTTGCTTGGGAAA-3’ |
| mouse *Mmp13* | 5’-AAGGGGATAACAGCCACTACAA-3’ | 5’-ACCAACATAAAAATTAAGCCAAATG-3’ |
| mouse β-actin | 5’-CATCCGTAAAGACCTCTATGCCAAC-3’ | 5’-ATGGAGCCACCGATCCACA-3’ |
| mouse *Gapdh* | 5’-GTGAAGGTCGGTGTGAACG-3’ | 5’-TCGCTCCTGGAAGATGGTG-3’ |
| Human GAPDH | 5’-CTCCTCCACCTTTGACGCTG-3’ | 5’-TCCTCTTGTGCTCTTGCTGG-3’ |
| *Ova* | 5’-AGAAATGTCCTTCAGCCAAGCTC-3’ | 5’-GCCCATAGCCATTAAGACAGATGTG-3’ |

**Supplementary Figures and Figure Legends**

**FIGURE S1 |** Characterization of CAVs generated using induction solutions with different concentrations of PFA. (a) Representative TEM images of CAVs obtained from induction solutions containing varying PFA concentrations. (b) Representative DLS profiles of CAVs produced with different PFA concentrations.

**FIGURE S2 |** Representative hydrodynamic size distribution of CAVs after 7 days of storage at 4°C.

**FIGURE S3 |** Schematic diagram illustrating the SPAAC reaction.

**FIGURE S4 |** Schematic of the preparation procedure for N_3_‑CAV.

**FIGURE S5 |** Rapid in vitro conjugation of N_3_‑CAV to Albumin-DSPE‑PEG‑DBCO‑pretreated cell surfaces via click chemistry. (a) Schematic illustrating the labeling of N_3_‑CAV with the lipophilic dye DiI. (b) Schematic of the cell‑labeling procedure and representative CLSM images: SVEC4-10 cells were pretreated with either albumin-DSPE-PEG or albumin-DSPE-PEG-DBCO for 24 h. After removing unbound complexes, the cells were incubated with N₃‑CAVs (red) for 5 min, washed to remove unbound vesicles, and then imaged. Nuclei were stained with Hoechst (blue). Scale bar = 20 μm.

**FIGURE S6 |** The modular aggregation system enables efficient LN targeting via click chemistry. (a) Schematic of the experimental workflow for evaluating lymphatic targeting efficiency. C57BL/6 mice were pre-treated with different components, followed by footpad injection of DiR-labeled N_3_-CAVs and subsequent IVIS imaging to quantify lymphatic drainage. (b) Representative whole‑body IVIS images (top) and corresponding ex vivo images of excised inguinal LNs (bottom) from C57BL/6 mice across different treatment groups. (c) Relative fluorescence density in inguinal LNs under different treatments, quantified from (b). Data are expressed as mean ± SEM. Statistical significance was determined by Student’s *t*-test. ***p <* 0.01.

**FIGURE S7 |** Synthesis and construction of CAV^OVA^. (a) Schematic of the design and construction of an OVA overexpression vector. (b) Schematic of the CAV^OVA^ preparation process. (c) Relative expression of *Ova* mRNA in HEK293T cells under indicated treatments. (d) Relative abundance of *Ova* mRNA in CAV from HEK293T cells under indicated treatments; CAV^empty^ refers to CAV produced by cells transfected with the empty vector. Data are expressed as mean ± SEM. Statistical significance was determined by one-way ANOVA with Tukey’s post hoc test. *****p* < 0.0001, ns, not significant.

**FIGURE S8 |** Efficient uptake of CAVs by DCs. (a) Schematic of CAVs co-cultured with DCs. (b) Representative CLSM images demonstrating CAVs uptake by DCs. DiI-labeled CAVs (red) and Hoechst-stained nuclei (blue) are shown. Scale bar = 20 μm.

**FIGURE S9 |** Size and weight of inguinal LNs following various treatments at day 3. (a) Representative digital photographs of the inguinal LNs from mice subjected to different treatments on day 3. (b) Inguinal LNs weights across treatment groups on day 3. Data are expressed as mean ± SEM. Statistical significance was determined by one-way ANOVA with Tukey’s post hoc test. **p* < 0.05, ***p* < 0.01, *****p* < 0.0001, ns, not significant.

**FIGURE S10 |** Super-resolution US imaging reveals vaccination-induced hemodynamic changes in LNs microvasculature. (a) Representative super-resolution US imaging of LNs before and after vaccination. Scale bar = 1 mm. (b-g) Quantitative hemodynamic analysis of the LNs microvasculature derived from (a): mean velocity (b), mean density (c), perfusion index (d), vessel ratio (e), curvature index (f), complexity level (g). Data are expressed as mean ± SEM. Statistical significance was determined by one-way ANOVA with Tukey’s post hoc test. **p* < 0.05, ***p* < 0.01, ns, not significant.

**FIGURE S11 |** LN volume expansion post-vaccination. Representative B‐mode US images of the dLNs (delineated by white dashed lines) over a 10-day period. Scale bar = 1 mm.

**FIGURE S12 |** Size and weight of inguinal LNs following various treatments on day 10. (a) Representative digital photographs of the inguinal LNs from mice subjected to different treatments on day 10. (b) Inguinal LNs weights across treatment groups on day 10. Data are expressed as mean ± SEM. Statistical significance was determined by one-way ANOVA with Tukey’s post hoc test. ns, not significant.

**FIGURE S13 |** DCs activation on day 10 post-vaccination. (a) Representative flow cytometric analysis of DC maturation markers in dLNs of C57BL/6 mice at 10 d post indicated treatments. (b-d) Quantification of the percentages of CD11c⁺CD80⁺CD86⁺ (b), CD11c⁺CD80⁺ (c), and CD11c⁺CD86⁺ (d) DC populations from (a). Data are expressed as mean ± SEM. Statistical significance was determined by one-way ANOVA with Tukey’s post hoc test. ns, not significant.

**FIGURE S14 |** CD8**^+^** T cells in dLNs on day 3 post-vaccination. (a) Representative flow cytometry analysis of CD8⁺ T cells in dLNs across treatment groups on day 3. (b) Quantification of the percentage of CD8**^+^** T cells from (a). Data are expressed as mean ± SEM. Statistical significance was determined by one-way ANOVA with Tukey’s post hoc test. ***p* < 0.01, *****p* < 0.0001, ns, not significant.

**FIGURE S15 |** Serum cytokine levels in mice following treatment. (a) Serum levels of TNF-α at 10 d post-treatment. (b) Serum levels of IL-6 at 10 d post-treatment. (c) Serum levels of IFN-γ at 3 d post-treatment. Data are expressed as mean ± SEM. Statistical significance was determined by one-way ANOVA with Tukey’s post hoc test. **p* < 0.05, ***p* < 0.01, ns, not significant.

**FIGURE S16 |** Representative hydrodynamic size distribution of N_3_-SCAV^H/R^ after 7 days of storage at 4°C in the dark.

**FIGURE S17 |** SenoVac combined with US effectively clears senescent cells in *p16-tdTomato* reporter mice. Abundance of senescent cells (red) in major organs (heart, liver, spleen, lung, kidney) across different treatment groups. Nuclei were counterstained with Hoechst (blue). Scale bar = 100 µm.

**FIGURE S18** | Abundant senescent cells in the aortic root in high-fat diet-fed ApoE^−/−^ mice. (a) Plaque localization in aortic roots from high-fat diet-fed ApoE^−/−^ mice was shown by ORO and H&E staining. (b) Immunofluorescence staining of P21 expression (in red) in the aortic sinus. Nuclei were stained with Hoechst (in blue).

**FIGURE S19 |** Quantification of the ORO-positive plaque area in the aortic sinus from Figure 6e. Data are expressed as mean ± SEM. Statistical significance was determined by one-way ANOVA with Tukey’s post hoc test. ***p* < 0.01, *****p* < 0.0001, ns, not significant.

**FIGURE S20 |** Aortic stiffness assessed by PWV in ApoE^−/−^ mice across treatment groups. (a) Schematic diagram of aortic arch PWV measurement in mice. (b) Statistical analysis of PWV in the aortic arch of ApoE^−/−^ mice under indicated treatments. Data are expressed as mean ± SEM. Statistical significance was determined by one-way ANOVA with Tukey’s post hoc test. ****p* < 0.001, *****p* < 0.0001, ns, not significant.

**FIGURE S21 |** Quantification of the P21-positive cells in the aortic sinus from Figure 7a. Data are expressed as mean ± SEM. Statistical significance was determined by one-way ANOVA with Tukey’s post hoc test. *****p* < 0.0001, ns, not significant.

**FIGURE S22 |** CD4⁺ and CD8⁺ T cell responses were assessed in ApoE^−/−^ mice at 16 weeks. (a) Flow cytometric analysis of splenic CD8^+^ and CD4⁺ T cells across treatment groups. (b-c) Quantification of CD8^+^ T cells (b) and CD4⁺ T cells (c) from (a). Data are expressed as mean ± SEM. Statistical significance was determined by one-way ANOVA with Tukey’s post hoc test. ns, not significant.

**FIGURE S23 |** Renal function in ApoE^−/−^ mice under various treatments. (a-c) Serum levels of UA (a), CREA (b), and BUN (c) across indicated groups. UA: uric acid; CREA: creatinine; BUN: blood urea nitrogen. Data are expressed as mean ± SEM. Statistical significance was determined by one-way ANOVA with Tukey’s post hoc test. ns, not significant.

**FIGURE S24 |** Liver function in ApoE^−/−^ mice under various treatments. (a-d) Liver function biomarkers in ApoE^−/−^ mice: levels of ALT (a), AST (b), TBIL (c), and ALB (d). ALT: alanine aminotransferase; AST: aspartate aminotransferase; TBIL: total bilirubin; ALB: albumin. Data are expressed as mean ± SEM. Statistical significance was determined by one-way ANOVA with Tukey’s post hoc test. **p* < 0.05, ns, not significant.

**FIGURE S25 |** SenoVac combined with US ameliorates senescence-associated fatty liver. (a) Representative B-mode US images of the liver and kidney from each group of mice. (b) Statistical analysis of the liver-to-kidney ratio across treatment groups. Data are expressed as mean ± SEM. Statistical significance was determined by one-way ANOVA with Tukey’s post hoc test. **p* < 0.05, *****p* < 0.0001, ns, not significant.

**FIGURE S26 |** Cardiac function in treated ApoE^−/−^ mice. (a) Representative M-mode and mitral inflow Doppler echocardiographic images from the indicated groups. (b-c) Quantification of systolic function parameters: left ventricular ejection fraction (EF) (b) and fractional shortening (FS) (c). (d) Quantification of the diastolic function parameter: E/A ratio. Data are expressed as mean ± SEM. Statistical significance was determined by one-way ANOVA with Tukey’s post hoc test. ns, not significant.

**FIGURE S27 |** Analysis of serum lipid metabolism in ApoE^−/−^ mice across indicated groups. (a-d) Measurements of serum lipids include LDL-C (a), HDL-C (b), TC (c), TG (d). LDL-C: low-density lipoprotein cholesterol; HDL-C: high-density lipoprotein cholesterol; TC: total cholesterol; TG: triglycerides. Data are expressed as mean ± SEM. Statistical significance was determined by one-way ANOVA with Tukey’s post hoc test. ns, not significant.
